# Bivalent single domain antibody constructs for effective neutralization of Venezuelan equine encephalitis

**DOI:** 10.1038/s41598-021-04434-x

**Published:** 2022-01-13

**Authors:** Jinny L. Liu, Dan Zabetakis, Christina L. Gardner, Crystal W. Burke, Pamela J. Glass, Emily M. Webb, Lisa C. Shriver-Lake, George P. Anderson, James Weger-Lucarelli, Ellen R. Goldman

**Affiliations:** 1 Center for Biomolecular Science and Engineering, U.S. Naval Research Laboratory, Washington, DC USA; 2 Virology Division, U.S. Army Medical Research Institute of Infectious Diseases, Ft. Detrick, MD USA; 3 Virginia Polytechnic Institute and State University, Blacksburg, VA USA

**Keywords:** Biochemistry, Biotechnology, Molecular engineering

## Abstract

Venezuelan equine encephalitis virus (VEEV) is a mosquito borne alphavirus which leads to high viremia in equines followed by lethal encephalitis and lateral spread to humans. In addition to naturally occurring outbreaks, VEEV is a potential biothreat agent with no approved human vaccine or therapeutic currently available. Single domain antibodies (sdAb), also known as nanobodies, have the potential to be effective therapeutic agents. Using an immune phage display library derived from a llama immunized with an equine vaccine that included inactivated VEEV, five sdAb sequence families were identified that showed varying ability to neutralize VEEV. One of the sequence families had been identified previously in selections against chikungunya virus, a related alphavirus of public health concern. A key advantage of sdAb is the ability to optimize properties such as neutralization capacity through protein engineering. Neutralization of VEEV was improved by two orders of magnitude by genetically linking sdAb. One of the bivalent constructs showed effective neutralization of both VEEV and chikungunya virus. Several of the bivalent constructs neutralized VEEV in cell-based assays with reductions in the number of plaques by 50% at protein concentrations of 1 ng/mL or lower, making future evaluation of their therapeutic potential compelling.

## Introduction

Venezuelan equine encephalitis virus (VEEV), a member of the *Alphavirus* genus of the *Togaviridae* family, is an important human and veterinary pathogen that is transmitted by mosquitoes. VEEV infections mainly target the central nervous system and lymphoid tissues causing severe encephalitis in equines and a spectrum of human diseases ranging from unapparent to acute encephalitis. While equine mortality rates are reported between 19 and 83%, infected humans usually develop a non-specific febrile illness, with neurological disease appearing in 4–14% of cases^1^. Furthermore, VEEV is highly infectious by aerosol inhalation in humans and other animals, rendering it a potential biothreat in addition to being a naturally occurring re-emerging disease^2^.

Veterinary vaccines based on inactivated virus are available, but currently there are no FDA-approved vaccines or therapeutics against VEEV for humans^3^. An attenuated strain of VEEV (TC-83), originally developed as a vaccine by the US Army^4^, has been used as a vaccine by laboratory workers and the US military. Although TC-83 is reasonably effective in preventing human disease, approximately 20% of recipients that receive the TC-83 vaccine fail to develop neutralizing antibodies, while between 15 to 37% develop febrile symptoms^4^.

Lacking an approved vaccine against VEEV, a prophylaxis and/or therapy that could effectively treat this serious and potentially fatal viral infection is desired. Towards the goal of developing therapeutics, monoclonal antibodies (mAbs) that show binding and neutralization of VEEV have been evaluated. MAbs have been shown to protect mice from aerosol challenge^5,6^ and recently, they have been demonstrated to protect non-human primates from severe disease, even when administered 48 h after aerosol exposure^7^. Although promising, traditional mAbs are large complex molecules that can be expensive to produce, and have short shelf lives unless maintained in cold storage. Due to their size, they fail to cross the blood brain barrier; thus, they are ineffective for treating encephalitic infections. In addition, mAbs are difficult to bioengineer to possess additional desired properties.

Single domain antibodies (sdAb, also known as nanobodies or VHH) are the variable domains derived from the unconventional heavy chain only antibodies found in camelids, and combine the specificity and affinity of conventional antibodies with the ability to be easily produced recombinantly and engineered towards specific applications^8–11^. Other advantages of sdAb include their small size, about 1/10 the size of conventional antibodies (~ 15 kDa versus ~ 150 kDa), their ability to refold and bind antigen after denaturation, and the ability to recognize hidden epitopes not recognized by conventional antibodies. SdAb also exhibit properties that are advantageous for therapeutics, including good tissue penetration in vivo, low immunogenicity, and the ability to tune the serum half-life through genetic fusions or PEGylation^12,13^. Additionally, sdAb have a proven safety profile: Ablynx, a Sanofi pharmaceutical company, currently has multiple sdAb in clinical development and the first product (caplacizumab for the treatment of acquired thrombotic thrombocytopenic purpura, TTP) was approved by the FDA in early February 2019^14^.

There are other examples of viral neutralizing sdAb^15,16^, including sdAb able to neutralize chikungunya, an alphavirus^17^. Several studies have shown that expressing sdAb as genetic fusions has led to improved neutralization^18,19^. For example, multimeric constructs, in which sdAb were genetically linked improved neutralization potencies up to 4,000-fold for Respiratory Syncytial Virus (RSV), 1,500-fold for Rabies virus and 75-fold for Influenza H5N1 and had potencies similar to or better than the best performing monoclonal antibodies^19^. The trivalent sdAb construct (ALX-0171) that inhibits RSV has also been successfully delivered by inhalation^20^, a route that may also prove valuable for treatment of VEEV transmitted via aerosols or to enhance uptake into the brain^21,22^.

In this report, we isolated several sequence families of anti-VEEV sdAb and evaluated their ability to inhibit VEEV infection in a plaque reduction neutralization test (PRNT). Constructs were prepared in which sdAb were genetically linked to themselves or a sdAb from a different sequence family, and compared to mixtures of standard sdAb in terms of neutralizing ability. We found that for some constructs, linking sdAb resulted in exponential gains in neutralization efficacy demonstrating the potential of these small, robust binding reagents for protection from VEEV.

## Materials and methods

### Reagents

Cloning enzymes (i.e. restriction endonucleases and ligase) were from New England Biolabs (Ipswich, MA, USA). Site-directed mutagenesis was performed using the QuikChange Lightning mutagenesis kit from Agilent (Santa Clara, CA, USA) following the manufacturer’s instructions. The Zoetis (Parsippany-Troy Hills, NJ, USA) West Nile innovator + VEWT equine vaccine was available from numerous US veterinary supply outlets. VEEV-TC-83 was from BEI Resources (Manassas, VA, USA). The BSL2 strain of chikungunya virus (CHIKV) strain 181/25 was kindly provided through the World Reference Center for Emerging Viruses and Arboviruses (WRCEVA, Galveston, TX). Our sdAb library used the pecan21 phage display vector^23^. DNA sequencing and gene synthesis were performed through Eurofins Genomics (Louisville, KY, USA).

Sucrose purified VEEV-TC-83 was exposed to10M rads of gamma–irradiation in a Model 109 Cobalt 60 irradiator. Inactivation of the gamma-irradiated VEEV-TC-83 was confirmed by serial blind passage in cell culture (Vero cells). The concentration of the irradiated VEEV-TC-83 preparation was determined by Pierce BCA protein Assay Kit (Cat#23,227) per manufacturer instructions. The absence of infectious virus in supernatant from passage 2 was confirmed in a standard plaque assay^7^.

### Llama immunizations and evaluation of immune response

Llama immunizations were performed by Triple J farms (Bellingham, WA, USA**)**. The immunization protocols used in this work were reviewed and specifically approved by the Triple J Farms Institutional Animal Care and Use Committee (IACUC). All methods were performed in accordance with the relevant guidelines and regulations. Three llamas (Whisper, Centavo, and Cowboy) were immunized with the West Nile Innovator + VEWT, an equine vaccine that includes inactivated VEEV. Centavo was immunized with the equine vaccine on days 0 and 14, with a bleed on day 28. Eight years prior, he had undergone a series of four immunizations with the same equine vaccine (immunizations on days 0, 21, 42, and 63). Both Whisper and Cowboy received a series of four immunizations on days 0, 14, 28, 42 with a bleed on day 56. For each animal, we isolated peripheral blood lymphocytes for the construction of phage display libraries, and saved the plasma to evaluate the immune response. Llama immune responses were evaluated by plaque reduction neutralization test (PRNT) described below.

### Library panning, and production of sdAb

We started with a previously described phage-display sdAb library derived from Centavo; this same library was used for selection of sdAb that recognize western equine encephalitis (WEEV)^24^. Three rounds of panning, using irradiated VEEV adsorbed to wells of 96-well plates, were carried out essentially as previously described^17^. For each round of panning we coated wells with 100 µL of 30 µg/mL irradiated VEEV to ensure that the wells were saturated with target antigen. A monoclonal phage enzyme linked immunosorbent assay (ELISA) was employed to identify positive clones after rounds 2 and 3. For the ELISA, wells of a 96 well plate were coated using 100 µL of 5 µg/mL. Positive clones were defined as having a signal to background ratio of over 2. Identified positive clones were re-streaked and subjected to DNA sequencing. We used the Multalin tool^25^ for sequence alignment when comparing the protein sequences of positive clones. The ANARCI tool was employed to number the amino acid sequence of the sdAb^26^, and complementarity determining regions (CDRs) were defined using the IMGT definitions^27^.

The coding sequences for the sdAb were each mobilized from the phage display vector into pET22b as *NcoI-NotI* fragments as described previously^28^. The sdAb expression plasmids were transformed into Tuner (DE3) for protein production and grown as previously described^24^. Purification of sdAb expressed from pET22b, the periplasmic expression vector, was achieved using an osmotic shock protocol, followed by immobilized metal affinity chromatography (IMAC) and fast protein liquid chromatography (FPLC) as described previously^29^. SdAb concentration was determined by UV absorption and stored at 4 °C or at -80 °C for long term storage.

### Circular Dichroism

Circular dichroism (CD) was performed in a Jasco J-815 Spectropolarimeter using a quartz cuvette with a 1 cm pathlength. Each sdAb was diluted to 15 µg/mL in distilled water. CD was measured at a wavelength between 200 and 210 nm as samples were heated from 25 to 85 °C at a rate of 2.5 °C/min followed by cooling at the same rate to determine the percent refolding after heat denaturation.

### Production of genetically-linked sdAb

Genetically-linked sdAb were prepared using the strategy described previously in which the first sdAb is flanked by *NcoI-NotI* restriction sites and the second sdAb flanked by *BamHI-XhoI* restriction sites with a “GGGGSGGGGSGGGGS” linker between them^30^. Bivalent sdAb were produced and purified using the same method used for standard sdAb described above.

### Plaque reduction neutralization test

SdAb were evaluated for neutralizing activity against VEEV (strain TC-83) and CHIKV (strain 181/25) in Vero cells. Two-fold serial dilutions of each sdAb were prepared starting at a concentration of 20 μg/mL. Dilutions were incubated with virus (~ 150–300 plaque forming units, PFU) overnight at 4 °C. Dilutions were plated in duplicate wells of a 6-well plate of Vero cells for 1.5 ± 0.5 h with gentle rocking every 15 min. Cells were covered with 0.6% agarose overlay in 1 × BME (Thermofisher) to each well and incubating for 17 h for TC-83 and 24 h for CHIKV at 37 °C in a humidified 5% CO_2_ atmosphere before the addition of the second 0.6% agarose overlay supplemented with neutral red vital stain. The plates were incubated at 37 °C for another 22–24 h before the transparent plaques were counted. The average of the duplicates was used to calculate 50% and 80% plaque reduction neutralization titer (PRNT_50_ and PRNT_80_) of each sdAb using the Matlab Curve Fitting tool. Values were determined by piecemeal interpolation with either a linear or exponential function, depending on the shape of the curve at the relevant point. When appropriate, the starting concentration of the sdAb construct was adjusted to include the dilutions ranging between PRNT_50_ and PRNT_80_. The average and standard deviation (STDEV) listed in the table were obtained from biological replicates. A similar method was used to determine the ability of standard and bivalent sdAb to neutralize wild type VEEV (strain TrD) and EEEV strain FL93-939^31^. The detailed protocol used to determine the ability of the sdAb to neutralize WEEV Imperial 181 strain^32^ (a gift from Dr. Aaron Brault) is presented in the supplemental material.

## Results and discussion

Three llamas, Whisper, Centavo, and Cowboy, were immunized with the West Nile Innovator + VEWT, an equine vaccine that includes inactivated VEEV. Plasma from each of the animals was evaluated to determine the ability to neutralize VEEV strain TC-83 as well as EEEV, WEEV, CHIKV, and West Nile virus (WNV). Results are reported in Table 1. Centavo, who had previously been immunized with a course of the West Nile Innovator + VEWT, had the strongest neutralization response of the three llamas; therefore, we chose to focus our effort on panning the phage-displayed sdAb library derived from this animal for the identification of VEEV-binding sdAb. It is unclear why the three animals had such different responses to this commercial vaccine, however Centavo likely benefited from previous inoculation. Interestingly Whisper, who showed a very high titer for West Nile virus, had been immunized with the commercial PreveNile and Recombitek equine vaccines for prevention of West Nile virus about the same time Centavo had been given his prior immunizations with West Nile Innovator + VEWT. We have observed that some animals generate more robust immune responses than others, and libraries prepared from animals possessing weak titers generally perform poorly.

**Table 1:** Neutralization of viruses by llama plasma.

	Centavo		Whisper		Cowboy	
	PRNT_80_	PRNT_50_	PRNT_80_	PRNT_50_	PRNT_80_	PRNT_50_
VEEV	1:320	1:1280	1:40	1:160	< 1:20	< 1:20
EEEV	1:320	1:640	1:80	1:320	< 1:20	< 1:20
WEEV	1:320	1:1280	1:20	1:160	< 1:20	< 1:20
CHIKV	1:20	1:40	< 1:20	< 1:20	1:20	1:40
WNV	1:2560	1:2560	1:2560	> 1:5120	1:160	1:320

**Figure 1:**
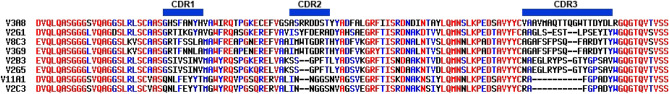
Amino acid sequences of representative sdAb selected for ability to bind irradiated VEEV-TC-83. Sequences are given in 1-letter amino acid code. Alignment was performed using Multalin^25^. Red indicates high homology, while lower homology is in blue. CDR regions are defined using the IMGT definitions^27^.

We panned the Centavo library using gamma-irradiation inactivated VEEV strain TC-83. Starting with approximately the same number of phage input for each of three rounds, we obtained increasingly greater numbers of output phage; with the second round output ~ 6 times greater than the first round, and the third round ~ 60 times higher than round 1. Although not the most robust enrichment, we proceeded to monoclonal phage ELISA for rounds 2 and 3. We examined 96 clones from each round and found 22 positives from round 2 and 32 from round 3. Sequencing of 40 positive clones identified five sequence families, three with at least 7 members, and two with only one member each. Interestingly, one of the sequence families (typified by V11A1 and V2C3, Fig. 1) was also isolated from a separate library derived from Centavo after immunization with chikungunya virus like particles about one year prior to the current immunization. Through that effort we had identified clone CC3, a sdAb that strongly neutralized CHIKV, and also neutralized VEEV^17,33^. While the same sequence family as CC3 was obtained from the new library, a sequence identical to CC3 was not (Supplemental Figure S1).

Eight sdAb, two representatives from each of the multi-member families as well as the two single-member sequence families, were transferred from the phage display vector to the pET22b expression vector for protein production (Fig. 1). In general, good protein yields were obtained from these clones as reported in Table 2. On examination of the sequences, we observed that V3A8, the poorest producing sdAb, had a non-conserved and unpaired cysteine in framework 2. In an effort to achieve better protein production, we performed site-directed mutagenesis to revert the cysteine to the conserved arginine to produce clone V3A8f. Unfortunately, this clone produced essentially identically to the original V3A8. Undeterred, a codon optimized version of V3A8f was synthesized which had a 3 to fourfold increase in protein production yielding 10.4 mg/L.

**Table 2:** Characteristics of isolated sdAb.

Clone	Tm (°C)	Refold %	Yield (mg/L)
V3A8	62	79	3.2
V2G1	73	99	13.3
V3G9	67	17	15.6
V8C3	67	13	6.6
V2B3	66	88	16.8
V2G5	65	54	18.4
V11A1	63	83	11.7
V2C3	64	71	19.6

The melting temperature and refolding ability of each sdAb was measured by circular dichroism and is reported in Table 2. Seven out of eight clones had melting temperatures between 62 and 67 °C with one clone, V2G1, melting at 73 °C. Likewise, most clones regained over 50% of their secondary structure after heat denaturation with the exception of the V8C3 and V3G9 sequence family which refolded poorly. Methods including the addition of negative charge and site-directed mutagenesis have been demonstrated to improve refolding ability and to raise melting temperature by as much as 10–20 °C, so the melting and refolding properties of these sdAb could potentially be improved by protein engineering, if desired^11^.

**Figure 2:**
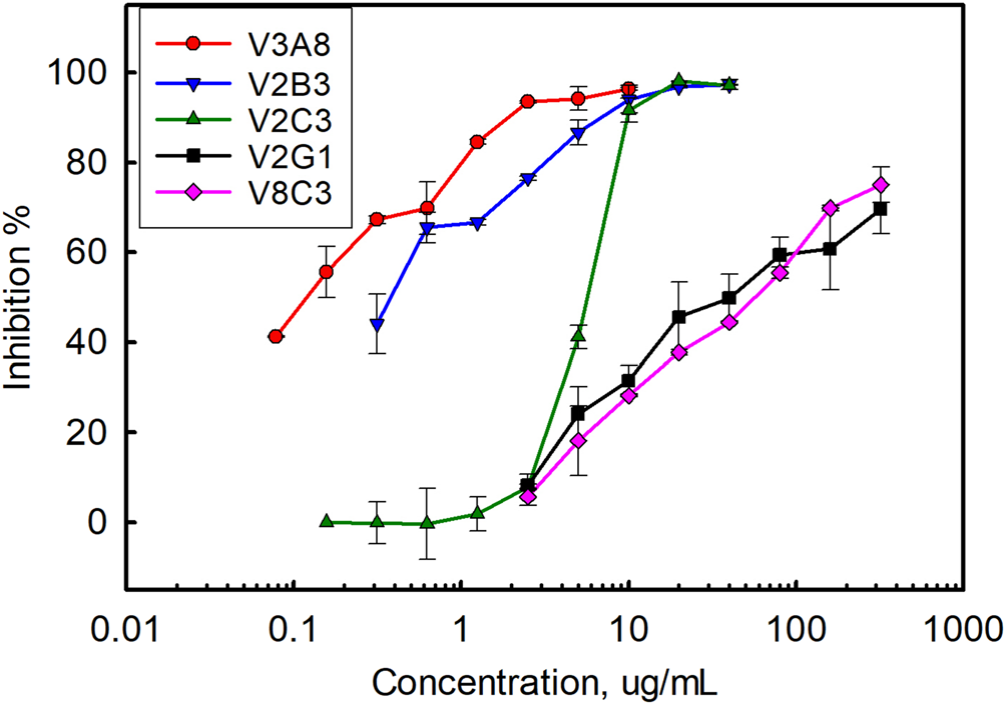
PRNT of representatives from each of the five anti- VEEV-TC-83 families. Data is shown from a representative set of neutralization experiments performed in duplicate. Error bars represent the standard error.

The eight clones were assessed by PRNT to determine their ability to neutralize VEEV strain TC-83. Several clones were also tested to determine if they were able to neutralize the virulent Trinidad Donkey (TrD) strain of VEEV. Results shown in Table 3 and Fig. 2 indicate that all families showed at least some ability to neutralize VEEV. In particular clones V3A8 and V2B3 showed the best neutralization, while V2G1 and V8C3 showed the weakest. Results from clones V2C3 and V11A1 were consistent with the previously isolated CC3 which we had measured a PRNT_50_ of 4.0 µg/mL and 1.9 µg/mL on the TC-83 vaccine strain and parental TrD strain respectively^33^.

**Table 3:** Neutralization of VEEV strains by standard sdAb.

Clone	PRNT_50_ TC-83 (µg/mL)	PRNT_80_ TC-83 (µg/mL)	PRNT_80_ TrD (µg/mL)
V3A8	0.16 ± 0.02	0.75 ± 0.20	Not done
V3A8f	0.22 ± 0.08	1.29 ± 0.01	50
V2G1	73.75 ± 48.58	506.30 ± 440.24	Not done
V3G9	351 ± 96.17	1570 ± 197.99	Not done
V8C3	40.40 ± 27.72	457.85 ± 5.02	> 50
V2B3	0.95 ± 0.42	2.67 ± 0.11	1.56
V2G5	3.94 ± 0.83	31.32 ± 7.95	Not done
V11A1	2.81 ± 0.78	8.70 ± 2.28	12.5
V2C3	5.48 ± 1.62	10.23 ± 1.98	25

Multiple studies have shown the advantage in using multivalent sdAb constructs for viral neutralization. Researchers have done this through genetically linked sdAb^19^, using tag-catcher systems^34^, and by fusions with an Fc region^35^. We chose to construct homo- and hetero-bivalent forms of the VEEV-binding sdAb by genetically linking sdAb through a flexible glycine-serine linker as detailed in the methods. Linked constructs were based on sdAb from four sequence families: V3A8f, V8C3, V2B3, and V2C3. To denote the linked constructs, we list the first sdAb followed by a hyphen and then the second sdAb. Protein yields of the bivalent constructs ranged from 2 to 9 mg/L.

**Figure 3:**
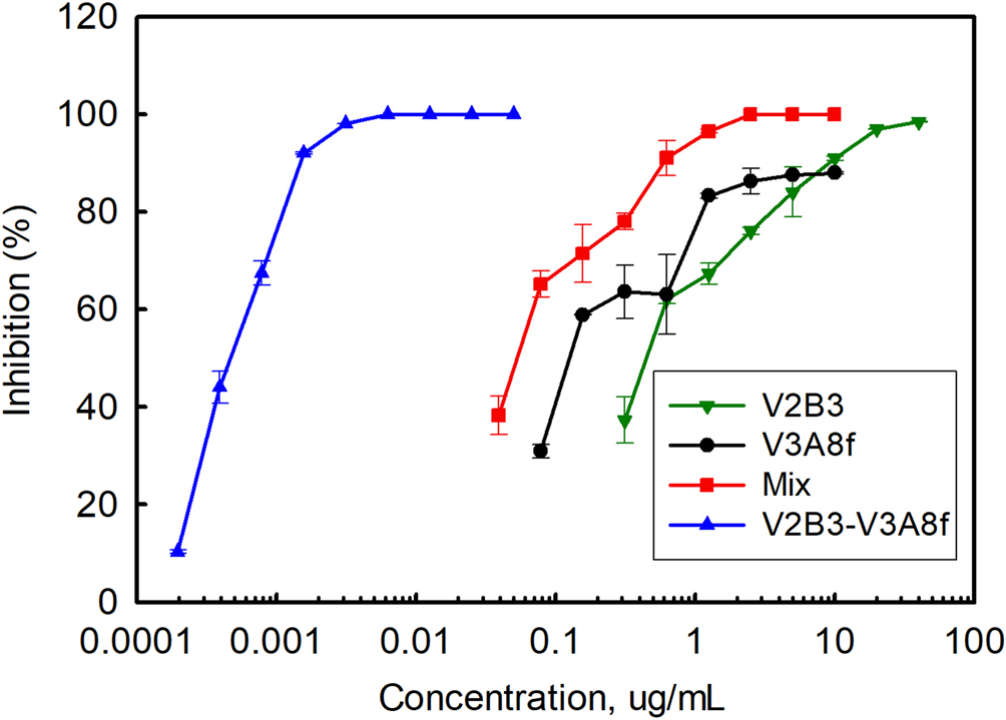
Representative PRNT data sets showing standard versus mixture versus linked sdAb. V2B3 and V3A8f are shown in green and black respectively. The mix of V2B3 and V3A8f is shown in red, and the linked V2B3-V3A8f is in blue. Each measurement was performed in duplicate, error bars represent the standard error.

To compare the ability of standard and bivalent constructs to neutralize VEEV-TC-83, we examined the neutralization ability of the bivalent sdAb versus mixtures of the standard sdAb. We also evaluated constructs where the two sdAb were linked in both orders. Data is shown in Table 4 and Fig. 3. We found that in each case an improvement was realized with the linked sdAb versus the mixture. The most potent were V3A8f-V2B3, and V2C3-V3A8f. Based on PRNT_50_, the V2C3-V3A8f construct showed almost 290-fold boost over the mixed sdAb. However, even the fusion of V3A8f with V8C3 showed ~ ninefold improvement over the mixture of standard constructs despite the fact that V8C3 by itself was a poor neutralizer. We did not see dramatic differences upon changing the order of the sdAb within the multimeric constructs, and chose to only study one orientation of the V8C3 and V3A8f mix.

**Table 4:** Neutralization of VEEV by standard sdAb or linked- sdAb (bold).

Table 4. PRNT for TC-83				
sdAb construct(s)	TC-83 PRNT_50_ (ng/mL)	PRNT_50_ fold-enhancement	TC-83 PRNT_80_ (ng/mL)	PRNT_80_ fold-enhancement
Mix of V2B3 + V3A8f	36.40 ± 3.82	–	204.3 ± 100.1	–
**V3A8f-V2B3**	0.76 ± 0.16*	**48**	**1.68 ± 0.74**	**122**
**V2B3-V3A8f**	**0.82 ± 0.17**	**44**	**1.63 ± 0.67**	**125**
V3A8f	216.0 ± 84.9	–	1285.0 ± 7.07	–
**V3A8f-V3A8f**	**0.81 ± 0.19**	**267**	**13.2 ± 16.8**	**97**
Mix of V8C3 + V3A8f	96.3 ± 9.48	–	481.5 ± 191.6	–
**V8C3-V3A8f**	**11.0 ± 3.61**	**9**	**23.6 ± 0.78**	**20**
Mix of V2C3 + V3A8f	204.0 ± 66.5	–	631.0 ± 29.7	–
**V3A8f-V2C3**	**1.65 ± 0.35**	**124**	**6.35 ± 0.64**	**99**
**V2C3-V3A8f**	**0.71 ± 0.24**	**287**	**1.45 ± 0.07**	**435**
V2B3	950 ± 420	–	2670 ± 110	–
**V2B3-V2B3**	**12.10 ± 2.83**	**78**	**40.4 ± 2.69**	**66**
Mix of V2C3 and CC3	1345 ± 521	–	4128 ± 2966	–
**CC3-V2C3**	**179.4 ± 41.9**	**7**	**482.9 ± 66.6**	**9**
V2C3	5480 ± 1620	–	10,230 ± 1980	–
CC3	2968 ± 34	–	8026 ± 1148	–
**V2C3-V2C3**	**157.0 ± 6.8**	**35**	**328.0 ± 157.8**	**31**

*STDEV.

The bivalent sdAb were also examined for their ability to neutralize the wild type TrD strain of VEEV. Results were consistent with the neutralization of VEEV-TC-83 and are reported in Table 5.

**Table 5:** Neutralization of VEEV-TrD.

sdAb construct	PRNT_50_ (μg/mL)	PRNT_80_ (μg/mL)	PRNT_90_ (μg/mL)
V2C3*	6.25	25	25
V3A8f	25	50	> 50
V3A8f-V3A8f*	0.024	0.195	0.781
V3A8f-V2B3	0.003	0.024	0.098
V2B3-V3A8f	0.003	0.006	0.006
V2C3-V3A8f	0.0015	0.003	0.006
V3A8f-V2C3	0.049	0.098	0.195
V2B3-V2B3	0.098	0.195	0.391
V8C3-V3A8f	0.049	0.098	0.195
V2B3*	0.781	6.25L	12.5
V8C3	> 50	> 50L	> 50L

*plaque size increased as concentration of antibody decreased.

Since we isolated sdAb, such as clone V2C3, which is in the same family as previously identified CHIKV-neutralizing sdAb CC3, we also tested a subset of standard and bivalent constructs for their ability to neutralize CHIKV. The PRNT_50_ and PRNT_80_ values are shown in Table 6. The V2C3 showed comparable neutralization of CHIKV as CC3; when V2C3 was expressed as a homobivalent construct or a heterobivalent construct with CC3, neutralization improved by ~ 5–sevenfold over the standard sdAb. We examined the heterobivalent construct of V2C3 with V3A8f as we hypothesized it has the potential to effectively neutralize both VEEV and CHIKV. Indeed the construct neutralizes both viruses with PRNT_50_ values ~ 2 ng/mL. However, while the linked V3A8f and V2C3 was much more effective at neutralizing VEEV than the mix of the standard sdAb constructs, it was not more effective than the mix at neutralizing CHIKV. When the ability of a mixture of V3A8f and V2B3 to neutralize CHIKV was compared to the bivalent version, the linked construct was no better, and potentially worse than the mixed sdAb clones.

**Table 6:** Neutralization of CHIKV with standard and bivalent sdAb.

sdAb construct(s)	PRNT_50_ (ng/mL)	PRNT_80_ (ng/mL)
CC3	2.2 ± 0.28*	11.9 ± 0.07
CC3-V2C3	0.30 ± 0.12	1.95 ± 0.35
V2C3-V2C3	0.43 ± 0.12	1.30 ± 0.28
V2C3	2.50 ± 1.27	12.8 ± 4.46
V3A8f-V2C3	2.30 ± 0.42	20.2 ± 3.18
Mix V3A8f + V2C3	2.10 ± 0.57	2.95 ± 0.21
V3A8f	646.5 ± 145.0	3159 ± 849.9
V2B3	633.00 ± 30.97	2376.00 ± 44.69
V3A8f-V2B3	2098.00 ± 2138.29	5247.50 ± 866.20
Mix V3A8f + V2B3	569.50 ± 104.65	2754.00 ± 104.70

*STDEV.

Selected standard and/or bivalent sdAb constructs were evaluated by PRNT to determine any cross reactivity with eastern equine encephalitis virus (EEEV) and western equine encephalitis virus (WEEV), two other new world alphaviruses. Preliminary studies showed that of the constructs tested, only CC3, V2C3-V2C3, and CC3-V2C3 neutralized EEEV with a PRNT_50_ of 25 or lower µg/mL (Supplemental Table S1). The bivalent constructs containing V3A8f weakly neutralized WEEV, but none neutralized greater than 30–45% at 50 µg/mL; the other linked constructs essentially did not neutralize WEEV at all. These results indicate that none of the sdAb constructs show appreciable cross-reactivity for these additional viruses. The E1 and E2 glycoproteins are the major antigenic proteins for which antibodies are produced^36^. Viruses with greater sequence homology in these glycoproteins have a higher likelihood of cross neutralization by antibodies that recognize conserved epitopes. A sequence comparison of the E1 glycoproteins across the alphaviruses found VEEV to be more closely related to EEEV (~ 60% sequence homology) than WEEV (~ 45% sequence homology)^37^. Based on the greater sequence homology between VEEV and EEEV, we expected to observe more cross neutralization with EEEV than WEEV.

We successfully met our goal of isolating sdAb that neutralized VEEV. Although it is likely that the sdAb bind to one of the envelope proteins, the exact target of these sdAb has yet to be determined. This is an interesting avenue for future experimentation which could include epitope mapping towards determining the mechanism of action of these sdAb.

The most potent of the bivalent sdAb constructs inhibited VEEV and prevented the infection of cells at low levels, thus offering the potential to be developed as therapeutics for the treatment of VEEV. In addition to providing a therapeutic for a biothreat of great concern, these sdAb may be of interest for treatment of both humans and horses during seasonal outbreaks of VEE. While horses are commonly immunized, there is currently no human vaccine, and should global warming expand the range of the mosquito vector the need for effective treatments could also likely increase. Combining the V3A8f clone with V2C3 produced a reagent with the potential to potently neutralize both VEEV and CHIKV. The next step will be to express the most promising structures in a format to provide increased serum half-life and test their effectiveness in a mouse model.

## Supplementary Information

Supplementary Information.

## Data Availability

Data is presented within the manuscript and the Supplemental Materials. The complete datasets generated during the current study are available from the corresponding author on reasonable request.
