# Supplementary material for: Bivalent single domain antibody constructs for effective neutralization of Venezuelan equine encephalitis
Source: Sci Rep. 2022 Jan 13;12:700. doi: 10.1038/s41598-021-04434-x (PMC8758676; doi:10.1038/s41598-021-04434-x)
Supplement: Supplementary file 1 — Supplementary Information. [file 41598_2021_4434_MOESM1_ESM.pdf]

Supplemental information

# Bivalent Single Domain Antibody Constructs for Effective Neutralization of Venezuelan Equine Encephalitis

Jinny L. Liu[1], Dan Zabetakis[1], Christina L. Gardner[2], Crystal W. Burke[2], Pamela J. Glass[2] Emily M. Webb[3], Lisa C. Shriver-Lake[1], George P. Anderson[1], James Weger-Lucarelli[3], and Ellen R. Goldman[1]*

[1]U.S. Naval Research Laboratory, Center for Biomolecular Science and Engineering, Washington, DC, USA

[2]U.S. Army Medical Research Institute of Infectious Diseases, Virology Division, Ft. Detrick, MD, USA

[3]Virginia Polytechnic Institute and State University, Blacksburg, VA, USA

*Corresponding author: Email: ellen.goldman@nrl.navy.mil, phone: 202-404-6052

Supplemental Figure S1

```
CC3        EVQLQASGGGSVQAGGSLRLSCVTSQNLFEYYTMGWYRQVPGSQRERVALINNGGSTVAGSVEGRFTISRDHAKNSVYLQMNYLKPEDSAVYYCRAFGPADYWGQGTQVTVSS
V11A1      DVQLQASGGGSVQAGGSLRLSCVASQNLFEYYTMGWYRQVPGSQRERVALINNGGSNVAGSVEGRFTISKDNAKNSIYLQMNNLKPEDSAVYYCRAFGPADYWGQGTQVTVSS
V2C3       DVQLQASGGGSVQAGGSLRLSCVASQNLFEYYTMGWYRQVPGSQRERVALINNGGSNVAGSVEGRFTISRDNAKNSIYLQMNNLKPEDSAVYYCRAFGPADYWGQGTQVTVSS
Consensus  #VQLQASGGGSVQAGGSLRLSCVaSQNLFEYYTMGWYRQVPGSQRERVALINNGGSnVAGSVEGRFTISrDnAKNS!YLQMNnLKPEDSAVYYCRAFGPADYWGQGTQVTVSS
```

Amino acid sequences of CC3, the previously isolated chikungunya virus-binding sdAb, compared to the sequences of V11A1 and V2C3, representative sdAb from one sequence family selected for their ability to bind irradiated VEEV-TC-83. Sequences are given in 1-letter amino acid code. Red indicates high homology.

Supplemental Table S1: Neutralization of EEEV-FL93-939

| | **$PRNT_{50}$ (µg/mL)** | **$PRNT_{80}$ (µg/mL)** | **$PRNT_{90}$ (µg/mL)** |
|---|---|---|---|
| V2C3* | >50 | 50 | >50 |
| V3A8f | >50 | >50 | >50 |
| V3A8f-V3A8f | >50 | >50 | >50 |
| V3A8f-V2B3 | >50 | >50 | >50 |
| V2B3-V3A8f | >50 | >50 | >50 |
| V2C3-V3A8f* | >50 | >50 | >50 |
| V3A8f-V2C3 | >50 | >50 | >50 |
| V2B3-V2B3* | >50 | >50 | >50 |
| V8C3-V3A8f* | >50 | >50 | >50 |
| V2B3 | >50 | >50 | >50 |
| V8C3 | >50 | >50 | >50 |
| CC3* | 25 | 50 | 50 |
| V2C3-V2C3* | 25 | 50 | 50 |
| CC3-V2C3* | 6.25 | 25 | 25 |

*plaque size increased as concentration of antibody decreased

Protocol for WEEV PRNT

1- Plate vero cells one day prior (targeted ~90% confluency)
2-Prepare two-fold serial dilutions of linked sdAb starting at 50 µg/ml in viral diluent (100 µl/well/linked sdAb concentration in 96-well U-bottom dilution plates)

*Viral diluent: RPMI-1640 media containing 25 mM HEPES, 1% BSA, 50 µg/mL Gentamicin, and 2.5 µg/mL Amphotericin B*

3-Prepare ~800 PFU/ml stock of WEEV (Targeting ≥40 plaques/well for 24-well plate)
4-Add WEEV PRNT stock to diluted linked sdAb (100 µl of 800 PFU virus stock added to 100 µl of diluted linked sdAb for 200 µl total)
5-Incubate linked sdAb/virus mixtures at 37°C for one hour
6-Add linked sdAb/virus mixture to vero cell monolayer (50 µl of mixture/well for 24-well plate)
7-Incubate cells at 37°C for one hour
8-Add 1.5% methylcellulose overlay to wells (500µl/well for 24-well plate)
9-Incubate plates at 37°C for 72 hours (specific to WEEV IMP 181 strain)
10-Gently decant methylcellulose overlay and fix plates with ~750 µl of 10% formalin for one hour
11-Decant 10% formalin, rinse plates with dH2O, and stain cells with 0.1% crystal violet solution
